# Supplementary material for: State-led agricultural subsidies drive monoculture cultivar cashew expansion in northern Western Ghats, India
Source: PLoS One. 2022 Jun 3;17(6):e0269092. doi: 10.1371/journal.pone.0269092 (PMC9165800; doi:10.1371/journal.pone.0269092)
Supplement: S1 Table — (DOCX) [file pone.0269092.s001.docx]

**S1 Table. An overview of Sawantwadi and Dodamarg *tehsils* (Data Sources: 10^th^ Agricultural Census, 2015-16; Census of India, 2011)**

| **Characteristics** | **Units** | **Sawantwadi** | **Dodamarg** |
| --- | --- | --- | --- |
| **General information** |  |  |  |
| Area | in km2 | 895.89 | 500.1 |
| Number of villages | - | 82 | 62 |
| Population | number of individuals | 147,466 | 48,904 |
| Literacy rate | % of total population | 86.71 | 75.37 |
| Population in rural areas | % of total population | 75.9 | 60 |
| Dominant ethnicity | % of total population | Hindu (92 %) | Hindu (96.21 %) |
| Number of households | - | 35,958 | 12,035 |
| **Farmers' information** |  |  |  |
| Marginal farmers (less than 1 ha) | % of total farmer population | 77.31 | 62.74 |
| Small farmers (1.00 to 2.00 ha) | % of total farmer population | 10.91 | 13.69 |
| Other farmers (above 2.00 ha) | % of total farmer population | 11.78 | 23.57 |
| Total land area owned by marginal farmers | % of total land area owned by all farmers | 21.4 | 8.56 |
| Total land area owned by small farmers | % of total land area owned by all farmers | 14.81 | 9.51 |
| Total land area owned by other farmers | % of total land area owned by all farmers | 63.79 | 81.92 |

Sources:

Tenth Agriculture Census of India, 2015-16, Department of Agriculture, Cooperation and Farmer Welfare, Ministry of Agriculture and Farmers Welfare. Government of India.

Census of India 2011. Provisional Population Totals-India data sheet. Registrar General, I., 2011. Office of the Registrar General Census Commissioner, India. Indian Census Bureau.
