# Supplementary material for: State-led agricultural subsidies drive monoculture cultivar cashew expansion in northern Western Ghats, India
Source: PLoS One. 2022 Jun 3;17(6):e0269092. doi: 10.1371/journal.pone.0269092 (PMC9165800; doi:10.1371/journal.pone.0269092)
Supplement: S2 Table — (DOCX) [file pone.0269092.s002.docx]

**S3 Table. Number of respondents interviewed per village.**

| **Tehsil** | **Village** | **Number of respondents** |
| --- | --- | --- |
| Dodamarg | A | 13 |
|  | B | 5 |
|  | C | 4 |
|  | D | 4 |
|  | E | 4 |
|  | F | 3 |
|  | G | 3 |
|  | H | 3 |
|  | I | 2 |
|  | J | 1 |
|  | K | 1 |
|  | L | 1 |
|  | M | 1 |
| Sawantwadi | N | 8 |
|  | O | 4 |
|  | P | 3 |
|  | Q | 2 |
|  | R | 1 |
|  | S | 1 |
|  | T | 1 |
| **Total:** | **20 villages** | **65** |
