# Supplementary material for: State-led agricultural subsidies drive monoculture cultivar cashew expansion in northern Western Ghats, India
Source: PLoS One. 2022 Jun 3;17(6):e0269092. doi: 10.1371/journal.pone.0269092 (PMC9165800; doi:10.1371/journal.pone.0269092)
Supplement: S3 Table — (DOCX) [file pone.0269092.s003.docx]

**S4 Table. Characterization of family and farm attributes across Small-, Medium-, and Large-holders.**

| **Number of respondents** | | | | | |  |
| --- | --- | --- | --- | --- | --- | --- |
| **Attributes** | **Marginal** | **Small** | **Semi-medium** | **Medium** | **Large** |  |
|  | **(< 1 ha)** | **(1-2 ha)** | **(2-4 ha)** | **(4-10 ha)** | **(≥ 10 ha)** |  |
|  | **(n=9)** | **(n=7)** | **(n=12)** | **(n = 17)** | **(n = 20)** |  |
| **Household attributes** |  |  |  |  |  |  |
| Age, range in years | 38 – 67 | 25 – 78 | 42 – 80 | 24 – 71 | 29 – 84 |  |
| Average age of the head cashew | 49.44 (9.89) | 57.71 (19.02) | 60.55 (10.59) | 46.35 (13.98) | 49.75 (12.53) |  |
| farmer in the household, in years (SD) |  |  |  |  |  |  |
| Average family size (SD) | 4.89 (4.31) | 3.71 (2.21) | 4.75 (4.61) | 6.53 (3.12) | 4.95 (2.63) |  |
|  |  |  |  |  |  |  |
| Average number of family | 1.63 (0.52) | 1.83 (0.41) | 1.75 (2.05) | 3.76 (3.07) | 2.2 (1.36) |  |
| members working in farms (SD) |  |  |  |  |  |  |
| Average farming experience, | 36.22 (14.43) | 35.79 (23.74) | 43.45 (21.86) | 38.59 (20.65) | 37.85 (15.18) |  |
| in years (SD) |  |  |  |  |  |  |
