# Supplementary material for: State-led agricultural subsidies drive monoculture cultivar cashew expansion in northern Western Ghats, India
Source: PLoS One. 2022 Jun 3;17(6):e0269092. doi: 10.1371/journal.pone.0269092 (PMC9165800; doi:10.1371/journal.pone.0269092)
Supplement: S4 Table — (DOCX) [file pone.0269092.s004.docx]

**S5 Table. Detailed typology of cashew farmers (n = 65) in the Sawantwadi-Dodamarg landscape, South Maharashtra, India.**

| **Sub-themes** | **Categories within sub-themes** | **Marginal** | **Small** | **Semi-medium** | **Medium** | **Large** |
| --- | --- | --- | --- | --- | --- | --- |
|  |  | **(< 1 ha)** | **(1 - 2 ha)** | **(2 - 4 ha)** | **( 4 - 10 ha)** | **(≥ 10 ha)** |
|  |  | **( n = 9)** | **( n = 7)** | **( n = 12)** | **( n = 17)** | **( n = 20)** |
| Cashew type planted | Cultivar only | 5 | 1 | 3 | 5 | 4 |
|  | Common only | 1 | 1 | 2 | 1 | 1 |
|  | Combination of cultivar and common | 3 | 5 | 5 | 10 | 15 |
|  | No response | 0 | 0 | 2 | 1 | 0 |
| Number of farmers that availed of subsidies | yes | 3 | 2 | 4 | 11 | 14 |
|  | no | 6 | 5 | 6 | 5 | 6 |
|  | tried unsuccessfully | 0 | 0 | 0 | 1 | 0 |
|  | no response | 0 | 0 | 0 | 0 | 0 |
| Pesticide usage | Chemical | 2 | 5 | 6 | 10 | 8 |
|  | Organic | 0 | 0 | 1 | 0 | 1 |
|  | none | 7 | 2 | 5 | 7 | 11 |
| Fertiliser usage | Chemical | 5 | 4 | 8 | 14 | 10 |
|  | Organic | 2 | 1 | 2 | 2 | 4 |
|  | none | 2 | 2 | 2 | 1 | 6 |
| Labour employed in farm | Local | 3 | 7 | 8 | 13 | 12 |
|  | Combination of local and non-local labour | 0 | 0 | 1 | 1 | 4 |
|  | No labour employed | 6 | 0 | 3 | 3 | 4 |
| Farm ownership status | Inherited | 6 | 6 | 10 | 15 | 16 |
|  | Bought | 2 | 1 | 0 | 1 | 2 |
|  | Inherited & bought | 0 | 0 | 0 | 1 | 2 |
|  | Bought & on lease | 0 | 0 | 1 | 0 | 0 |
|  | Community land | 0 | 0 | 1 | 0 | 0 |
|  | Govt. provided | 1 | 0 | 0 | 0 | 0 |
| Average price received per kilogram of cashew nuts in 2018 (in INR) | - | 167.81 (16.66) | 169.64 (8.95) | 156.50 (24.48) | 168.82 (10.08) | 170.65 (12.22) |
| Sale of cashew nuts produce | Personally | 5 | 4 | 4 | 9 | 11 |
|  | Middlemen/ merchants | 4 | 2 | 7 | 6 | 6 |
|  | combination of both | 0 | 1 | 0 | 2 | 3 |
|  | No response | 0 | 0 | 1 | 0 | 0 |
| Prices received fair or not | Fair | 3 | 5 | 7 | 12 | 14 |
|  | Not fair | 4 | 1 | 2 | 3 | 4 |
|  | At the mercy of market fluctuations | 1 | 1 | 1 | 1 | 1 |
|  | No response | 1 | 0 | 2 | 1 | 1 |
| Number of farmers who are members of co-operatives | - | 3 | 1 | 1 | 2 | 7 |
