# Supplementary material for: State-led agricultural subsidies drive monoculture cultivar cashew expansion in northern Western Ghats, India
Source: PLoS One. 2022 Jun 3;17(6):e0269092. doi: 10.1371/journal.pone.0269092 (PMC9165800; doi:10.1371/journal.pone.0269092)
Supplement: S1 Appendix — (DOCX) [file pone.0269092.s005.docx]

**S2 Appendix. The survey design which was used to interview cashew farmers in Sawantwadi and Dodamarg.**

**Semi structured questionnaire for interviews**

*Good morning/afternoon Sir/Madam. My name is ___ and I am conducting surveys on cashew farming practices as part of a PhD research project on how private land such as private forests and cashew farms are managed. The survey is supported by Nanyang Technological University, Singapore. Your responses shall be kept confidential. You may choose to not respond to any question you wish. There would be no monetary remuneration for being a respondent for this interview. We would appreciate your support which is crucial for this survey. There would be no wrong or correct answer. Would you like to be interviewed?*

*If no, thank the participant and stop.*

*If yes, continue.*

*Do you own or farm a cashew farm?*

*If no, thank the participant and stop.*

*If yes, then continue.*

| Start time: | End Time: |
| --- | --- |
| Surveyor: | Date: |
| Sub-district: | Village: |

a. Name:

b. Age:

c. How long have you lived here- since what year?

**Farm plot information**

d. How many plots do you own?

| Sr, no. | Question | Plot 1 | Plot 2 | Plot 3 | Plot 4 |
| --- | --- | --- | --- | --- | --- |
| - | How large are the plot(s)? |  |  |  |  |
| 1. | What crops do you grow? |  |  |  |  |
| 2. | How long have you been growing these crops? In years |  |  |  |  |
| 3. | Are the plots leased/ bought/ inherited/community land? |  |  |  |  |
| 4. | How is farm segregated? (Fenced, bamboo, Other) |  |  |  |  |
| 5. | How much yield per plot? |  |  |  |  |
| 6. | Are the cashew on your farm(s) *jungli* (common) or *kalma* (cultivar)? How many of each? |  |  |  |  |
| 7. | Which *kalma* variety? |  |  |  |  |
| 8. | How much yield from each type? |  |  |  |  |
| 9. | What rate did the yield sell at per kilo? |  |  |  |  |
| 10. | Are you happy with both/either? |  |  |  |  |
| 11. | How many trees of *jungli*/ *kalma* are planted? |  |  |  |  |
| 12. | Do you use pesticide for both *jungli*/*kalma*? |  |  |  |  |
| 13. | Which ones do you use? |  |  |  |  |
| 14. | How much quantity of these are used per tree? |  |  |  |  |
| 15. | Do you use fertiliser for both *jungli* / *kalma*? |  |  |  |  |
| 16. | Which ones do you use? |  |  |  |  |
| 17. | How much quantity of these are used per tree? |  |  |  |  |
| 18. | Do you use protective gear while using fertiliser/ pesticide? | s |  |  |  |
| 19. | Do you employ labour from other places? How many? |  |  |  |  |
| 20. | Which places do labourers come from? |  |  |  |  |
| 21. | What season do they come in? |  |  |  |  |
| 22. | How much is the yield per plot? |  |  |  |  |
| 23. | Do you sell *bondu* (cashew apple)? At what rate? |  |  |  |  |
| 24. | Has the govt. ever helped you with farming subsidies? |  |  |  |  |
| 25. | If yes, what kind? |  |  |  |  |
| 26. | Any shade trees grown on the farm? |  |  |  |  |
| 27. | Which ones? |  |  |  |  |
| 28. | How many of each? |  |  |  |  |
| 29. | Is your house close to the farm _____ or do you use a separate farmhouse _________? |  |  |  |  |
| 30. | Do you send the produce or does a merchant buy it? |  |  |  |  |
| 31. | Which market is the produce taken to? |  |  |  |  |
| 32. | How much does sending produce cost? |  |  |  |  |
| 33. | How do you send it? Tempo __ car__ bus___ factory_ vehicle __ by themselves _ |  |  |  |  |
| 34. | When do you begin harvest? _________ When do you end the harvest? __________ |  |  |  |  |
| 35. | How much is spent in farm maintenance? |  |  |  |  |
| 36. | What land use is the farm surrounded by? |  |  |  |  |
| 37. | How close is the forest to plot? |  |  |  |  |
| 38. | How does it matter in terms of yield or expenditure, if forest is close-by? |  |  |  |  |
| 39. | Do you face crop loss due to animals? Which ones? |  |  |  |  |
| 40. | How much damage in yield?  Damage to full trees or just fruits / nuts? |  |  |  |  |
| 41. | How frequently do you see wild animals? |  |  |  |  |
| 42. | How often do people visit the plot ? |  |  |  |  |
| 43. | How dense is the plot vegetation? |  |  |  |  |
| 44. | How often is the plot cleared/ weeded? |  |  |  |  |

**Family information**

1. How many members are there in the household currently?

2. How many members are under 18 years of age?

3. How many male and female members: male: female:

4. How many members work in the farms?

5. Are there any secondary sources of occupation?

| Family member |  |  |  |  |  |  |
| --- | --- | --- | --- | --- | --- | --- |
| Sec. occupation |  |  |  |  |  |  |

5. Does cashew income come in constantly or once a year?

**Market information & Human Wildlife Interactions**

1. Any issues faced in transport?

2. Is the market price fair to you?

3. Are you a part of any farmers’ co-operative?

4. Are there any facilities for credit/ short term loans/ capital that you avail of? What are they?

5. How do you deal with wild animals on your farm?

6. Did you face loss due to animals / disease, and apply for compensation?

7. If you applied, then how much compensation did you receive in the past?

8. Was it an easy process?

9. If not, why so? Why do you think the FD don’t or can’t help?

10. Do you want the crop loss and human-wildlife interactions to stop?

11. Do you think this situation is better/ worse than 5 and 10 years back?

12. Do you think any spaces in your village are very frequently used by wild animals?

13. Do you own *malki* (private) jungle?

14. How big is it?

15. How are you dependent on it?

16. How often do you visit it?

*Thank the respondent for their co-operation. Answer any queries they may have.*
